# Redefining the concept of interoperability: A multidimensional approach for the energy sector

**DOI:** 10.12688/openreseurope.20755.2

**Published:** 2026-05-20

**Authors:** Joep van Genuchten, Laia Guitart, Carlos Ayon Mac Gregor, René Kuchenbuch, Thomas I. Strasser

**Affiliations:** 1 Contractor at EPRI Europe DAC, Dublin, D04 V2N9, Ireland; 2 E.DSO – European Distribution System Operators, Brussels, 1040, Belgium; 3 B.A.U.M. Consult GmbH, Berlin, 12435, Germany; 4 OFFIS eV, Oldenburg, Lower Saxony, 26121, Germany; 5 AIT Austrian Institute of Technology, Vienna, Vienna, 1210, Austria

**Keywords:** Energy transition, governance, interoperability, semantic change, Smart Grid Architecture Model, socio-technical systems, standards, system integration.

## Abstract

**Background:**

Interoperability is a foundational concept in the development of modern energy systems, yet its traditional definitions, centered on technical compatibility and information exchange, fail to capture the complexity of the energy transition. As energy infrastructure becomes increasingly decentralized, digitalized, and governed by diverse actors, a broader understanding of interoperability is required.

**Methods:**

This study draws on insights from the European Horizon Europe project int:net, and conducts a comprehensive analysis of 40 definitions of interoperability across sectors, languages, and periods. The definitions were evaluated using both lexical and temporal approaches and mapped against the Smart Grid Architecture Model, which was extended with a sixth “Framework Layer” to incorporate governance and institutional dimensions.

**Results:**

The findings reveal that existing definitions predominantly emphasize technical aspects, with limited attention paid to organizational, social, and regulatory factors. The analysis demonstrates a semantic shift in the term ‘interoperability’ over time and highlights the need for a multidimensional approach. A revised definition is proposed, framing interoperability as an emergent property of systems shaped by shared standards, collaboration, and contextual alignment across technical and nontechnical domains.

**Conclusions:**

This study presents a holistic redefinition of interoperability tailored to the energy transition, supported by an extended Smart Grid Architecture Model framework. It offers practical recommendations for implementation, including stakeholder engagement, flexible standardization, and interdisciplinary capacity-building. This approach aims to support the development of resilient, inclusive, future-ready energy systems.

**List of abbreviations**
BY-NC
: Attribution-NonCommercial (Creative Commons license).
CC: Creative Commons.
CGMES: Common Grid Model Exchange Standard.
EC: European Commission.
EIF: European Interoperability Framework.
ENTSO-E
: European Network of Transmission System Operators for Electricity.
EU: European Union.
GWAC: GridWise Architecture Council.
ICT: Information and Communication Technology.
IEC: International Electrotechnical Commission.
IEEE: Institute of Electrical and Electronics Engineers.
M/490: European Mandate M/490 (related to Smart Grid standardisation).
NATO: North Atlantic Treaty Organisation.
SCADA: Supervisory Control and Data Acquisition.
SGAM: Smart Grid Architecture Model.
UK: United Kingdom.

## 1. Introduction

Interoperability is a crucial concept in today’s interconnected world, yet its current interpretation often falls short in the context of the energy transition. The shift towards decarbonized energy infrastructure introduces novel challenges that call for a re-examination of the definition and implementation of interoperability. In the energy sector, the notion of interoperability has evolved significantly, particularly with the rise of digital control and smart grid technologies. Early efforts to achieve system compatibility began in the 1980s with the integration of Supervisory Control and Data Acquisition (SCADA) systems, highlighting the need for seamless communication between infrastructure components from different vendors. ^1^

The emergence of the smart grid concept in the late 1990s underscored the importance of standardized protocols for integrating information and communication technologies with traditional energy systems. ^2, 3^ By the early 2000s, formal efforts to define and implement interoperability standards gained momentum and laid the groundwork for a more interconnected energy ecosystem, emphasizing the necessity of consistent standards to accommodate renewable energy integration and digital advancements that challenge the traditional notions of interoperability and calling for a redefinition. ^4^

The primary motivation for redefining interoperability stems from insights gained during the European int:net project, ^5^ which revealed critical limitations in the existing definitions. Traditional definitions predominantly focus on technical aspects, such as the ability of hardware or software to share information, but overlook equally important dimensions, such as human factors, organizational structures, and the impact of changing environments. By redefining interoperability, the project seeks to build a holistic understanding that includes technical, social, organizational, and environmental factors, creating a comprehensive and adaptable concept that reflects all elements surrounding interoperability. An examination of the existing interoperability definitions and the Smart Grid Architecture Model (SGAM) framework ^6^ as an established tool in the energy sector for evaluating interoperability efforts shows that an interoperability definition that comprehensively considers all challenges is needed. This will effectively support the energy sector in addressing the current and future challenges.

This paper examines existing definitions of interoperability across various domains, evaluates them considering insights from the int:net project, and proposes an updated definition tailored to the challenges of energy transition. In doing so, it also acknowledges the broader context of an increasingly digitalized world, where interconnected systems span multiple sectors and domains. These developments highlight the need for a more holistic and adaptive understanding of interoperability that transcends traditional sectoral boundaries.

The remainder of this paper is organized as follows. Section 2 discusses the implications of poorly defined interoperability terms, whereas Section 3 examines the triangle of meaning and the changing meanings of words. An assessment of existing interoperability definitions is presented in Section 4, followed by a discussion of domain-specific perspectives on interoperability using the SGAM framework in Section 5. A new definition of interoperability, along with recommendations for its implementation, is presented in Section 6. Finally, the paper concludes with preliminary guidelines for operationalizing this conceptual framework in Section 7.

## 2. Impact of ambiguous terms

The term interoperability is widely used across technical, social, and policy domains yet often lacks a clear and consistent definition. This ambiguity can significantly affect system design, policy implementation, and societal outcomes. The following sections examine how unclear interpretations of interoperability impact technical development, policy coherence, and social progress, underscoring the need for shared understanding to support effective collaboration and innovation.

### 2.1 Technical implications

A clear and widely accepted understanding of interoperability is critical for guiding the design of technology. Effective solutions require precise requirements. What functions should the system perform? How should they respond to specific conditions? Without clear definitions of the key terms, technical teams create incompatible or inefficient systems. Models such as SGAM and the GridWise Interoperability Context-Setting Framework ^7^ offer structured approaches to define interoperability in technical contexts. These frameworks are widely used in the energy sector to design and integrate systems across various domains. They provide a foundation for identifying the technical dimensions of interoperability and are explored in greater detail in Section 5. A key consequence of a poorly defined concept or divergent understanding of interoperability is the development of incompatible solutions, even when frameworks, such as SGAM, exist to guide the process. Failure to adopt such models leads to technical interoperability challenges, as designers make inconsistent assumptions about how systems should interact.

### 2.2 Policy implications

When policymakers use the term ‘interoperability’ without providing a clear and explicit definition, it can lead to significant challenges. On the one hand, policies built on narrow definitions may enforce rigid technical solutions such as standards that might stifle innovation and limit the emergence of new technologies. On the other hand, it can lead to fragmentation and operational inefficiencies, such as resource waste or delayed implementation of new technologies in the energy system, hindering the energy transition.

If the term is not correctly defined, stakeholders may interpret the concept differently, resulting in fragmented, uncoordinated, and often conflicting efforts to achieve interoperability. This lack of clarity undermines collaboration and hampers the effectiveness of collective effort. For example, consider a city that aims to enhance its public transportation system by ensuring seamless connections between buses and trains. If policymakers fail to specify what they mean by “interoperability,” some stakeholders might prioritize getting additional buses, while others may focus on updating schedules or improving communication between services. Without a unified understanding of the term, these initiatives are unlikely to be coordinated effectively, and the overarching goal of an integrated transportation network will not be achieved.

### 2.3 Social implications

The importance of an open energy system and of how interoperability (or lack thereof ) extends beyond technical and policy considerations relates to the social implications of using the term “interoperability” in a poorly defined way, which may result in long-term challenges, such as vendor lock-in and increased costs for society. When interoperability is not clearly defined or enforced through open standards, systems are often developed using proprietary technologies that are difficult to integrate. This results in vendor lock-in, where users are tied to specific vendors for upgrades, maintenance, and future system expansion. In the energy sector, this can significantly limit flexibility, inflate costs, and inhibit innovation by preventing the adoption of more efficient and competitive solutions.

From a societal perspective, this lack of flexibility leads to inefficient use of public resources and can delay the adoption of clean energy technologies, as their integration with existing infrastructure becomes more complicated and costly. For example, if a region’s smart grid infrastructure is not interoperable with newer solar or battery technologies from other providers, these technologies may be excluded, despite their potential social and environmental benefits.

## 3. Language, meaning, and transformation

As language evolves, so too does the meaning of key terms, particularly in dynamic and technologically driven fields, such as energy systems. A common question arises: can the definition of a term, such as interoperability, simply be changed? If so, how can such a redefinition contribute to greater clarity, rather than confusion? To address this issue, it is useful to consider how meaning is constructed and how it changes over time.

### 3.1 The triangle of meaning

**Figure 1.**
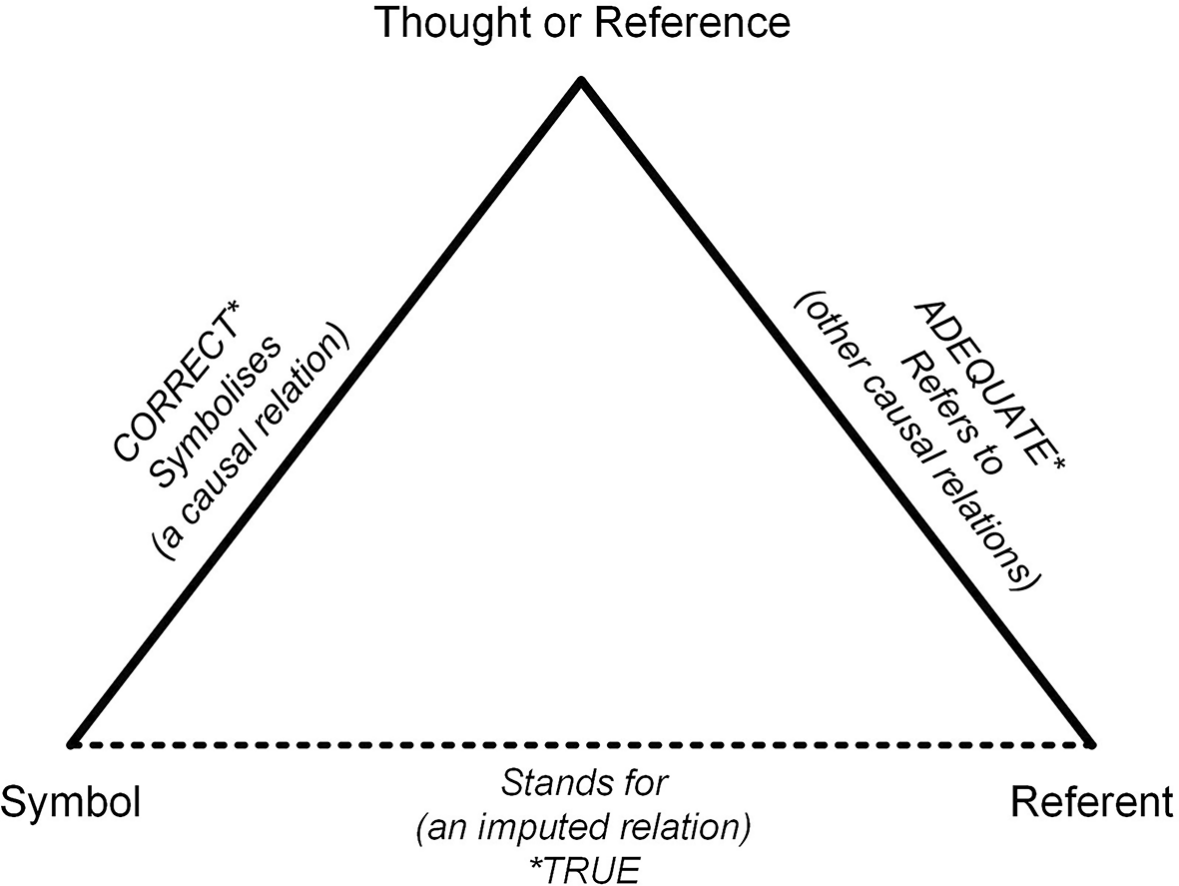
The triangle of meaning. ^8^

The “Triangle of Meaning” was introduced by Ogden and Richards in 1923. ^8^ It is a widely used model for understanding language functions. This illustrates the relationship between the three elements (see Figure 1):

• *“Symbols”:* These are words (written or spoken), pictures, or anything that tries to share meaning (i.e., interoperability).
• *“Referent”:* This refers to the real things out there in the world that we want to talk about.
• *“Thought”:* This is our mental image of what is happening in reality.

This model emphasizes that the connection between a symbol (word) and a referent (thing) is not direct, but mediated by thought. Miscommunication occurs when different people attach different concepts to the same word, which is particularly common with technical or abstract terms, such as interoperability. Similarly, different terms can be used to describe the same underlying concepts across languages or disciplines. If two people have different thoughts, but use the same words, communication can be difficult. The other way around: If someone speaks French and another person speaks Dutch, they will have trouble understanding each other, even though they are thinking about the same thing.

The Triangle of Meaning illustrates that on the one hand, words can carry different meanings depending on the context, and on the other hand, individuals may use different words to refer to the same concept. This idea is particularly relevant to the present work, as it is argued that the term ‘interoperability’ has a slightly different meaning today than it may have in the past. This phenomenon, in which the meaning of a word shifts over time, is referred to as ‘semantic change.’

### 3.2 Semantic change in practice

Language adapts over time to accommodate new realities, often through a process called semantic change, in which the meanings of words evolve while retaining a link to their original usage. A common example is the use of the pronouns ‘they’ and ‘them.’ Historically used only as a third-person plural, these pronouns have come to include gender-neutral singular use, reflecting a shift in societal understanding of gender identity. Another example is the word’ computer, which once described a job: someone who performed calculations. As the device we now call ‘computer’ was invented and became common, the job disappeared, and people needed a new word for these calculating machines. Thus, with time, the meaning of the term shifted to referring to electronic devices that perform computational tasks. Therefore, the meaning of ‘computer’ has changed to refer to the devices we use today. In this case, new things came to exist and we needed words to talk about them.

These examples illustrate that changes in meaning often arise not from linguistic manipulation, but from the need to describe new realities more accurately. Similarly, as the energy sector becomes more decentralized, digitalized, and data-driven, the traditional concept of interoperability, which focuses on hardware compatibility, no longer captures the full scope of requirements. The term must evolve to reflect this reality.

Recognizing this process legitimizes the effort to redefine interoperability in this study. Rather than destabilizing communication, a reasoned update to the term’s meaning, grounded in collective experience and practical needs, can enhance shared understanding and support more effective collaboration across disciplines and sectors.

## 4. Assessment of existing definitions

To critically reflect on how interoperability is currently understood, this section analyzes a broad set of existing definitions collected from different sectors, languages, and periods. The aim is to identify recurring patterns in terminology, trace changes in emphasis over time, and assess whether these definitions adequately reflect the complexity and requirements of modern systems, particularly within the context of energy transition. First, it assesses the common terminology used in the different definitions to identify which concepts are most strongly and consistently associated with the term. It then shows how the meaning of interoperability has evolved. This analysis lays the groundwork for a revised, broader definition of interoperability that better aligns with the current and future needs in the subsequent sections.

### 4.1 Method

To examine how the concept of ‘interoperability’ is defined across various contexts, this study collected definitions from a diverse range of sources, including documents in multiple languages. The non-English definitions were translated into English to ensure consistency. In total, 40 definitions were analyzed using two complementary approaches.

• *Lexical Approach:* A word frequency analysis was conducted to identify the most used terms across all definitions. A word cloud was generated to visualize these terms based on the assumption that frequently occurring words are more strongly associated with the concept and reflect a shared understanding.
• *Temporal Approach:* Building on lexical analysis, this approach also considers the publication dates of definitions. By examining how the usage of key terms has evolved, this analysis provides insights into how the meaning of *interoperability* has shifted, highlighting patterns of semantic change.

While conducting the analysis, editorial decisions were made. These choices can be categorized into three types.

1.
*Plurals, conjugations, and other spelling and grammatical variations*

To address this, we decided to

– Count plural nouns as singular and default to UK English spelling convention, for example: the words ‘organizations’, organizations’, ‘organization,’ and ‘organization’ were counted together and reported under’ organization.’
– Transform verbs into (singular) nouns, for example: ‘facilitate’ became ‘facilitation.’
– Transform adjectives into nouns, for example: ‘Seamless’ became ‘seamlessness.’

2.
*Combining words that are frequently used together*

Examples include:

– ‘Without restriction,’ transformed to ‘seamlessness.’
– ‘Two or more,’ transformed to ‘variety.’

3.
*Combine words that have very similar meanings*

This was the most subjective data-cleaning effort.

– All counts for ‘data’ and ‘information’ were combined and reported under ‘information.’
– All counts for ‘without restriction’ and ‘seamlessly’ combined and reported under ‘seamlessness.’

### 4.2 Commonly used terms

**Figure 2.**
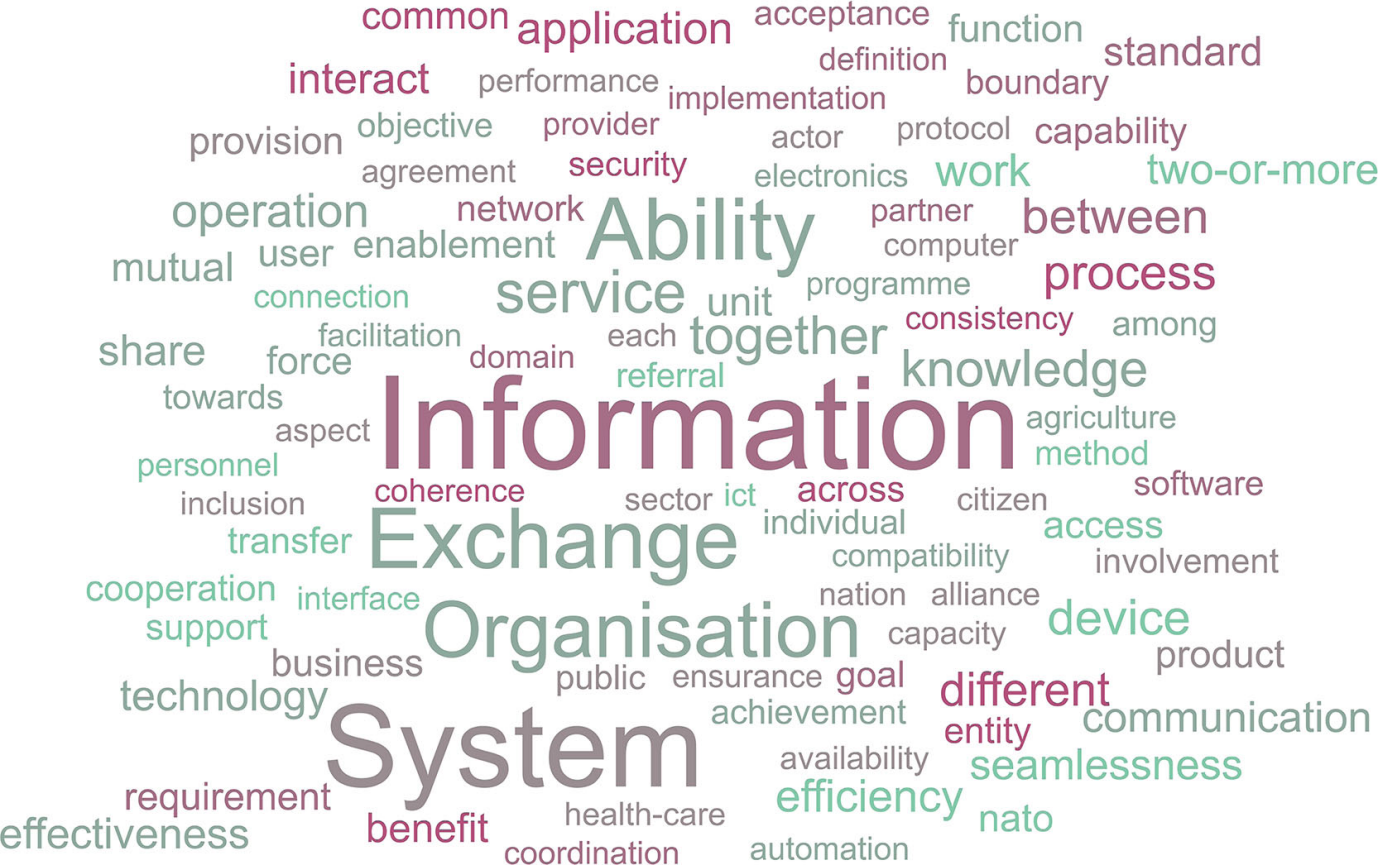
Word cloud based on the gathered definitions.

To gain an initial overview of how the concept of interoperability is commonly described, a word-frequency analysis of the definitions was conducted. This approach helps to highlight the most frequently occurring terms, offering insight into which concepts and ideas are most strongly associated with interoperability across contexts. To visually represent the prominence of these terms, a word cloud was created ( Figure 2). The use of a word cloud serves two key purposes. First, it provides an accessible and immediate visual summary of the most common language used. Second, it helps identify patterns or dominant themes that may not be as apparent through raw frequency tables alone. In this way, the word cloud acts as both a summary and discovery tool, setting the stage for a deeper temporal analysis in the following section.

**Figure 3.**
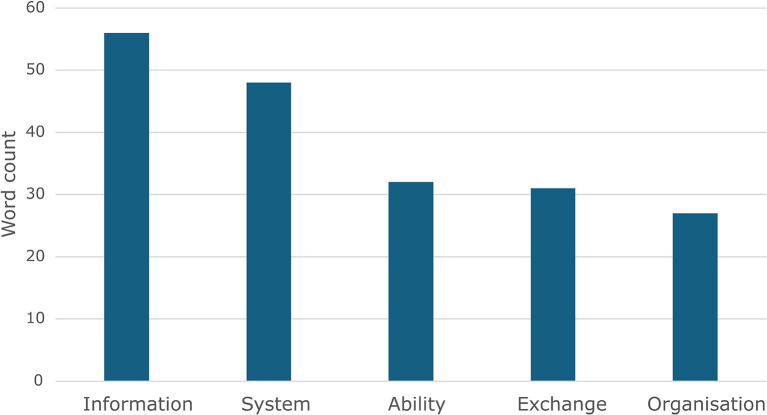
Bar graph depicting the word count of the five most common terms (note: data was transformed into information).

The word cloud representation suggests that the terms depicted in Figure 3 are the most widely understood to form part of the definition of interoperability (with the vertical axis representing the frequency by counting the occurrences of the most widely used terms).

A typical example of how the terms in the figure above are used together to define interoperability is the definition commonly used by IEEE.

*“The ability of two or more systems to exchange information and use the information that has been exchanged mutually”.* ^9^

This definition contains (the top) four of the five most-used terms. A typical example of a definition that uses ‘organization’ comes from the European Commission (EC):

*“Interoperability is the ability of organisations to interact towards mutually beneficial goals, involving the sharing of information and knowledge between these organisations, through the business processes they support, by means of the exchange of data between their ICT systems”.* ^10^

These definitions, and indeed most of the definitions that we found, seem to emphasize the ability to exchange information between systems and organizations. One set of definitions takes a slightly broader approach. These definitions are derived from the military. A telling example comes from the North Atlantic Treaty Organization (NATO):

*“Interoperability is the ability to act together coherently, effectively, and efficiently, to achieve Allied tactical, operational and strategic objectives. It comprises the human, technological and procedural dimension. [...]”* ^11^

While this definition still acknowledges technology as a factor, it places much more emphasis on the goal of what NATO is aiming to achieve with ‘interoperability’ and identifies ‘human, technological, and procedural dimensions. In many ways, this definition aligns better with the full scope of the SGAM framework (see Section 5 for further analysis) and the experiences from the int:net project than the general trend that someone can observe, which places a lot of emphasis on information exchange.

### 4.3 Temporal analysis

**Figure 4.**
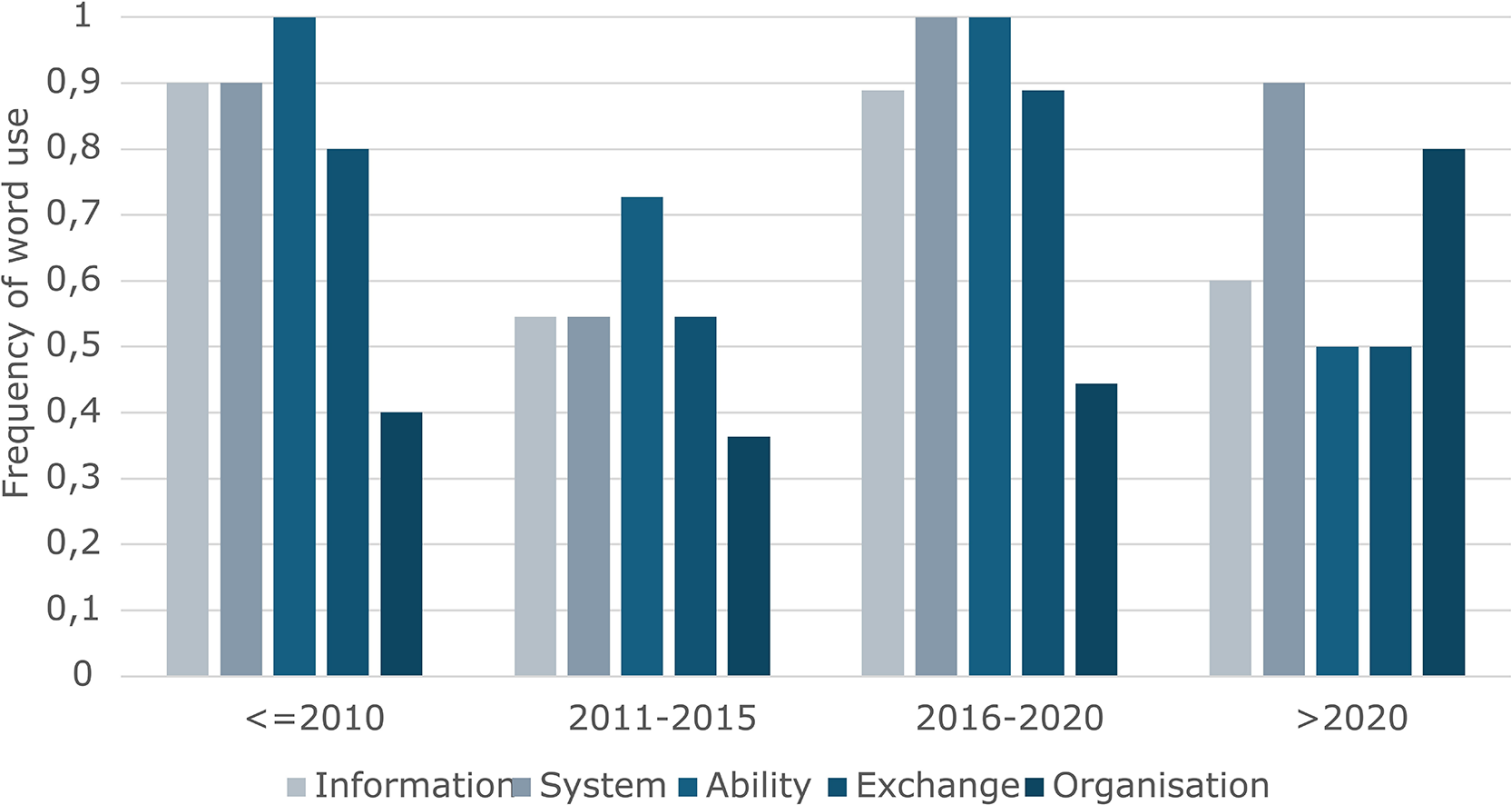
Usage of the five most common terms across periods.

To explore how the understanding of interoperability has evolved, a temporal analysis of the five most frequently used terms (i) ‘information’, (ii) ‘system’, (iii) ‘ability’, (iv) ‘exchange’, and (v) ‘organisation’ was carried out. We examined these terms across four periods: (i) before 2010, (ii) 2011–2015, (iii) 2016–2020, and (iv) after 2020. Figure 4 (with the vertical axis representing the frequency of word use, in percent, and the horizontal axis representing each of the five most common terms for each period) illustrates notable shifts in term usage over time:

• ‘Information’ and ‘system’ have consistently been among the most used terms across all periods, indicating their foundational role in the definition of interoperability. However, the usage of information slightly decreased in the most recent period (> 2020), suggesting a possible shift in focus.
• ‘Ability’ saw its lowest relative frequency between 2011 and 2015, and after 2020, but otherwise showed consistent use. This is possibly related to an attempt to concretize the term, often shown to fit the perspective of the entity developing the definition.
• ‘Exchange’ is often used before 2010, and between 2016 and 2020, otherwise decreasing and being substituted by phrases like ‘work together,’ ‘provide services,’ ‘efficiently share,’ ‘cooperate and make,’ ‘connect and communicate with.’ This could indicate a need for concretization, potentially linked to a shift in focus, that is, towards organizations instead of technology.
• ‘Organization’ had a usage of around 40% in the first three time periods (i.e., before 2020), but increased notably after 2020. This trend may reflect an increasing recognition of the role of institutions, governance, and collaborative structures in achieving interoperability.

This temporal lens provides insights into how the concept of interoperability has been framed differently over time, with shifts that may correspond to technological developments, policy changes, or evolving academic and industry priorities. Considering the small sample size (i.e., the number of definitions assessed), the encountered variability leads to the conclusion that the word ‘organization’ shows the most (or only) relevant shift.

The temporal trends identified suggest a shift in the conceptualization of interoperability. While early definitions focused predominantly on the exchange of ‘information’ between ‘systems,’ contemporary definitions increasingly incorporate terms such as ‘organization,’ indicating a broader understanding. This evolution reflects the growing recognition that interoperability is not solely a technical challenge, but also a socio-organizational one.

Technical interoperability, which is typically concerned with seamless operation and data exchange between systems, remains a foundational element. However, real-world implementations increasingly demonstrate that technical compatibility alone is insufficient to ensure effective interoperability. The growing prominence of terms like ‘organization’ in recent definitions reflects this broader understanding and a shift in the meaning of words. These terms highlight the importance of capabilities within and between institutions, shared standards and protocols, and the trust and coordination required among stakeholders. Moreover, interoperability must account for legal and policy differences, all of which require active management through governance structures and collaborative communities.

Considering these insights, there is a clear need to refine the definition of interoperability. A revised definition should integrate not only the technical requirements for system interaction, but also the organizational, institutional, and governance dimensions that underpin the interoperability context.

### 4.4 Consistency and change

Having carefully analyzed the 40 definitions of interoperability, it was noted that paying attention to the perspective of specific organizations led to additional insights. While some organizations show a consistent use of the definition, others seem to foster inconsistency in the usage of the term. For example, within the same standardization body (e.g., IEEE), three definitions of interoperability are consistently used:

• *“The ability of two or more systems or components to exchange information and to use the information that has been exchanged”*. ^12^
• *“The ability of two or more systems to exchange information and use the information that has been exchanged mutually”*. ^9^
• *“The ability of two or more systems or elements to exchange information and to use the information that has been exchanged”*. ^13^

These definitions show slight differences. In the second definition, the word ‘components’ was removed, while in the third, it was substituted by ‘elements.’ The second definition is found under a guide specifically drafted for a system environment, potentially explaining the change in the definition. A consistent understanding of the term across documents facilitates common understanding across working groups. The opposite effect can be seen in definitions obtained from the European Union (EU) in documents related to the European Interoperability Framework (EIF), the electricity market, and the public sector (correspondingly, in order of appearance):

• *“The ability of organizations to interact towards mutually beneficial goals, involving the sharing of information and knowledge between these organizations, through the business processes they support, by means of the exchange of data between their ICT systems.”* ^10^
• *“The ability of two or more energy or communication networks, systems, devices, applications or components to interwork to exchange and use information in order to perform required functions.”* ^14^
• *“Public sector interoperability is the ability of administrations to cooperate and make public services function across borders, sectors and organisational boundaries.”* ^15^

While the regulation from the EIF ^10^ provides a broad definition that may fit the context of different sectors, the definitions originating from the electricity market regulation and the press release on the Interoperable Europe Act (related to the public sector) specifically state context-related concepts in their definitions. Moreover, while electricity market regulation makes a clear emphasis on technical actors, the public sector definition clearly emphasizes the boundaries that it aims to integrate, providing insight into the challenges and opportunities of each sector, and potentially justifying the usefulness of having different definitions under different contexts.

## 5. Interoperability across domains via SGAM

**Figure 5.**
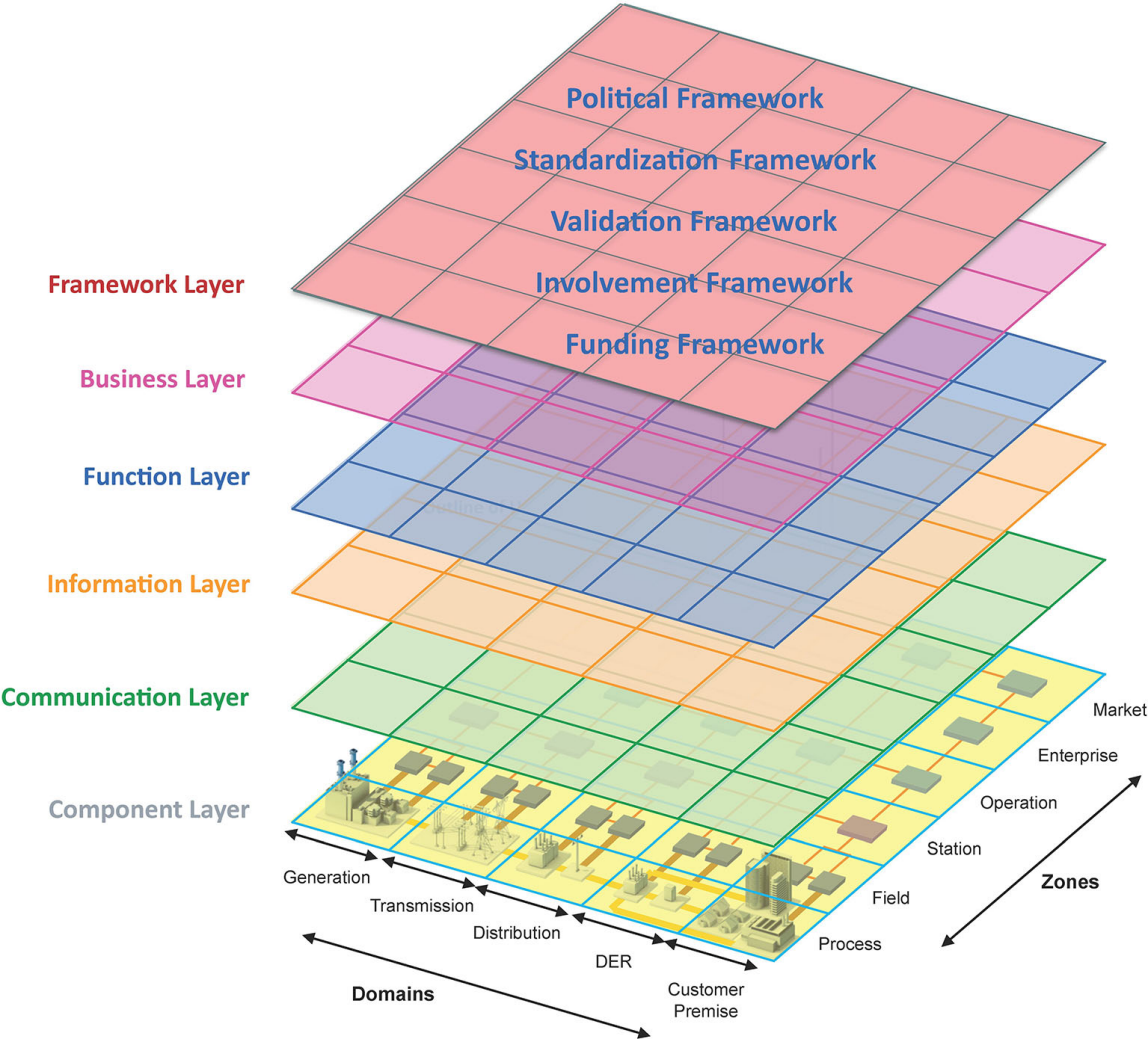
SGAM framework including the proposed sixth “Framework Layer”. ^19, 20^

In Section 4, the definition of interoperability is addressed from a linguistic perspective, focusing on the experiences from the int:net project. This European initiative addresses the development, testing, and deployment of interoperable energy services to further pave the path for a carbon-free European society by 2050, which led to the proposed redefinition of the concept. ^16^ One tool from the energy sector for discussing interoperability challenges is the SGAM framework (developed via the M/490 EU mandate), ^17, 18^ which was further developed both conceptually and in terms of its application as part of the int:net project. ^19^ This framework uses categories from the GWAC Interoperability Context-Setting Framework, ^7^ which can be considered domain-independent. The int:net project uses the SGAM Framework as a baseline and has identified the need to develop an additional layer, namely the “Framework Layer,” which is intended to address the governance and/or governmental aspects (in particular, the influence on the legislative and spatial boundaries) of interoperability. ^19, 20^ An illustration of the extended SGAM Framework, including an additional Framework Layer, is shown in Figure 5.

The SGAM framework serves as a fundamental basis for discussing and addressing interoperability challenges within the energy sector. However, not every interoperability definition covers all aspects outlined in the framework’s interoperability categories. By analysing the various definitions in relation to specific interoperability challenges, this study aims to clarify which definitions comprehensively address the relevant aspects. The existing interoperability challenges were therefore examined according to these definitions to support the development of a refined and more holistic interoperability definition. Each definition was classified according to its relevance to each SGAM layer, including the extended sixth “Framework Layer.” The following categories and classification schemes were used for this purpose:

• *Direct mentioning/strong:* This definition is compliant with the SGAM Layers’ visions for
  – Component Layer: The relevant actors, both technical and organizational, and their role in ensuring interoperability, are identified.
  – Communication Layer: The importance of communication, including the communication approach, protocols, standards, and their role in enabling interoperability, is explicitly stated and well-defined.
  – Information Layer: Information and data exchange, with a focus on ensuring interoperability, are directly addressed and clearly defined.
  – Function Layer: Functional aspects, use case-related objectives, and their impact on interoperability are clearly outlined.
  – Business Layer: Business objectives, business processes, and interoperability across organizational boundaries are highlighted.
  – Framework Layer: Governance, political, and regulatory aspects and how they influence interoperability boundaries are addressed.
• *Indirect mentioning/weak:* Indirect reference to the interoperability challenge in the definition, rather weak naming or implicatory.
• *No mentioning/at all:* The Interoperability challenge has not been addressed at all and is not included.

Consequently, numerical scores can be assigned to each definition based on the identified categories. For each interoperability layer, a score can be awarded. A ***direct mentioning*** or strong reference results in a full point, an ***indirect mentioning*** or weaker reference results in half a point, and ***no mentioning*** (no reference) results in zero points. The previously described criteria were used to code and evaluate each definition accordingly. Each definition can achieve a maximum of six points corresponding to the six interoperability layers.

**Figure 6.**
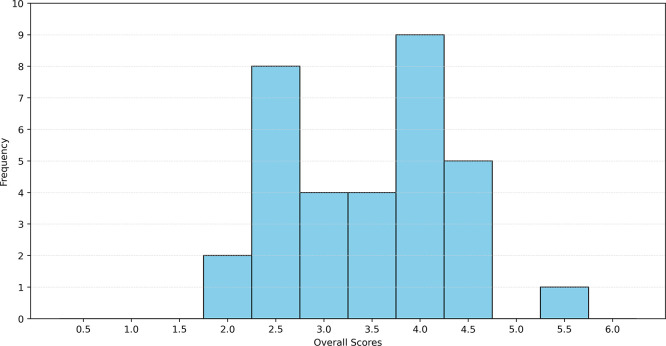
Score distribution for addressing interoperability challenges (based on SGAM) in selected definitions.

The results of the classification of the interoperability definitions for each layer are shown in Figure 6. Quantitative analysis revealed that most definitions strongly emphasize the exchange of information and communication aspects. Notably, all analyzed definitions reference either technical or community/institutional actors. However, the simultaneous consideration of both technical and organizational actors, as exemplified by the SGAM component layer, is not always observed.

Furthermore, a trend emerges concerning the level of interoperability: as the interoperability level increases within the SGAM framework, direct references to specific layers become less frequent, whereas indirect or absent references occur more often. This trend is particularly pronounced in the context of the framework layers. This also shows that there is a link between domain-related definitions and the frequency of reference to the Framework Layer.

**Figure 7.**
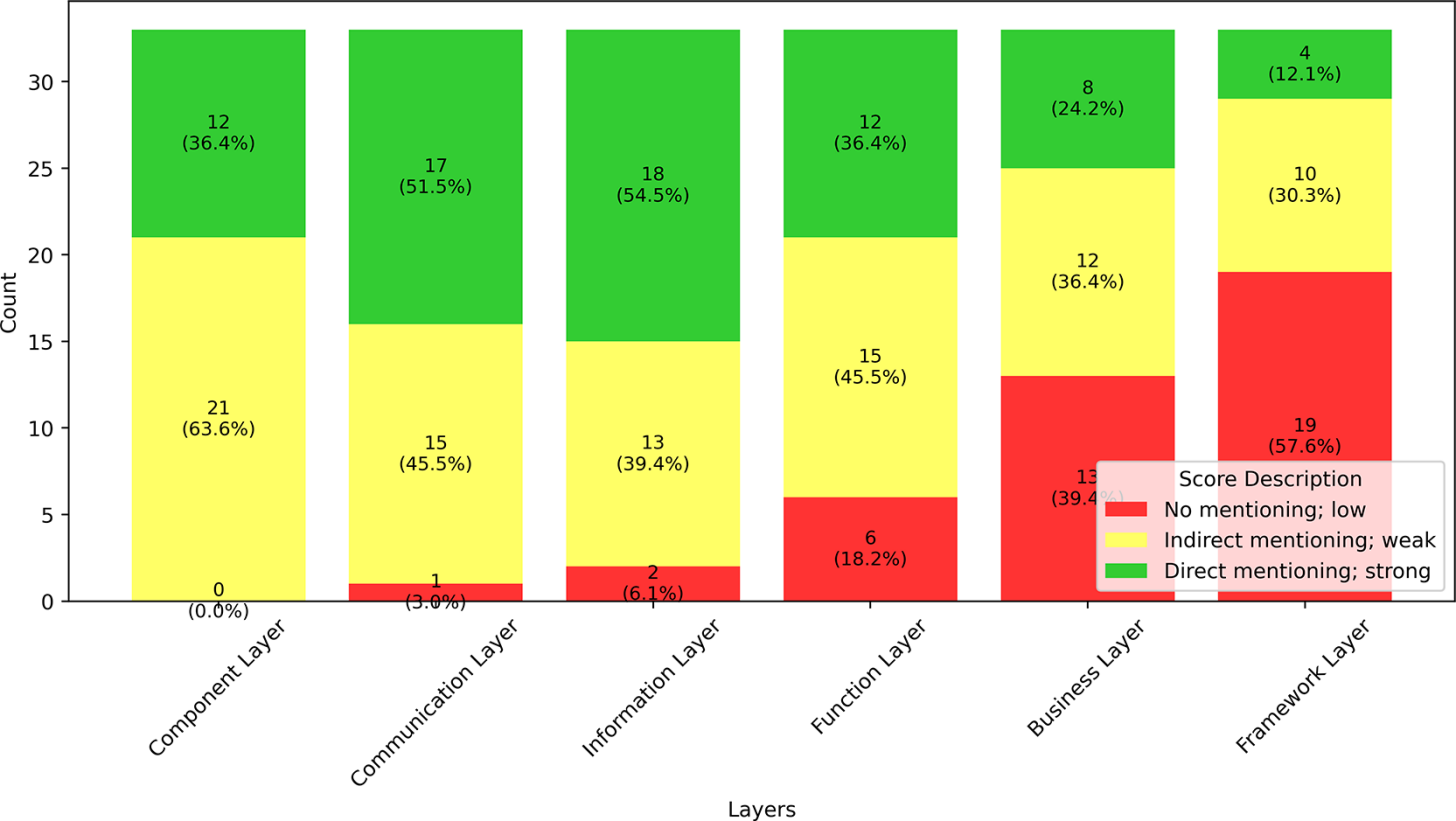
Distribution of the scores between the different SGAM interoperability layers.

The analysis revealed that both the mean and median values of the dataset were 3.5 (with a standard deviation of 0.87), assuming that explicit naming per layer results in one point, implicit naming results in 0.5, and no naming results in 0 points. The histogram of the data is shown in Figure 7. A maximum value of six was not attained, which implies that the definition accounts for all interoperability challenges according to the SGAM model. The Spanish interoperability definition achieved a value of 5.5 and can be considered domain-independent. It should be noted that aspects of the Framework Layer were not considered.

According to the Ibero American interoperability strategy, which most fully addresses SGAM layers in its definition, ^21^

*“Interoperability is understood as the ability of disparate and diverse organisations and systems to interact with agreed and common goals and with the aim of mutual benefit. Interaction implies that the organisations involved share information and knowledge through their business processes by exchanging data between their respective information and communications technology systems.”*

To derive our definition, it is therefore essential to address each interoperability challenge in int:net (selected using the SGAM framework), particularly the efforts related to the Framework Layer and associated topics such as regulation, governance, and funding. When examining the different SGAM layers, the lower three layers (component, communication, and information) are more oriented in individual systems, whereas the upper three layers are more oriented in the context. Figure 7 shows a clear jump in the fraction of definitions that have no mention of the qualities that concern the respective layer. This suggests that the authors of those definitions place more emphasis on the individual components than on the context. Focusing on the individual components naturally emphasizes the technical aspects of interoperability, leaving the social, organizational and systemic dimensions that were found during the int:net project underrepresented.

These results are also reflected in the results of the linguistic analysis (see Section 4). The terms information, system, and exchange are among the most frequently used terms in definitions. The more high-level terms (actor, capability, business, etc.) were used less frequently, which suggests a connection with the decreasing number of direct mentions as we moved to higher layers.

This section demonstrates how int:net contributes to a more nuanced understanding of interoperability in the energy sector by extending a state-of-the-art framework for considering interoperability in Europe, SGAM. Through the classification of existing interoperability definitions according to the SGAM layers, expanded by the proposed Framework Layer, it becomes clear that current definitions tend to focus heavily on technical aspects, such as communication and information exchange, while neglecting contextual elements, such as governance and regulation. This omission is especially evident in the upper SGAM layers, with the Framework Layer often receiving no direct mention. The analysis highlights a persistent gap in addressing interoperability challenges at the governance and institutional levels, underscoring the need for a broader and more integrative definition. The insights gained from the int:net project, particularly the emphasis on the Framework Layer, form a critical foundation for the redefinition of interoperability proposed in the following section.

## 6. New definition and implementation guidance

This section presents an updated definition of interoperability, informed by prior analysis and experience in int:net, to address the complexity of modern interconnected systems in the energy sector. It also provides recommendations for its application across various domains and organizations.

### 6.1 Proposed definition of interoperability

**Figure 8.**
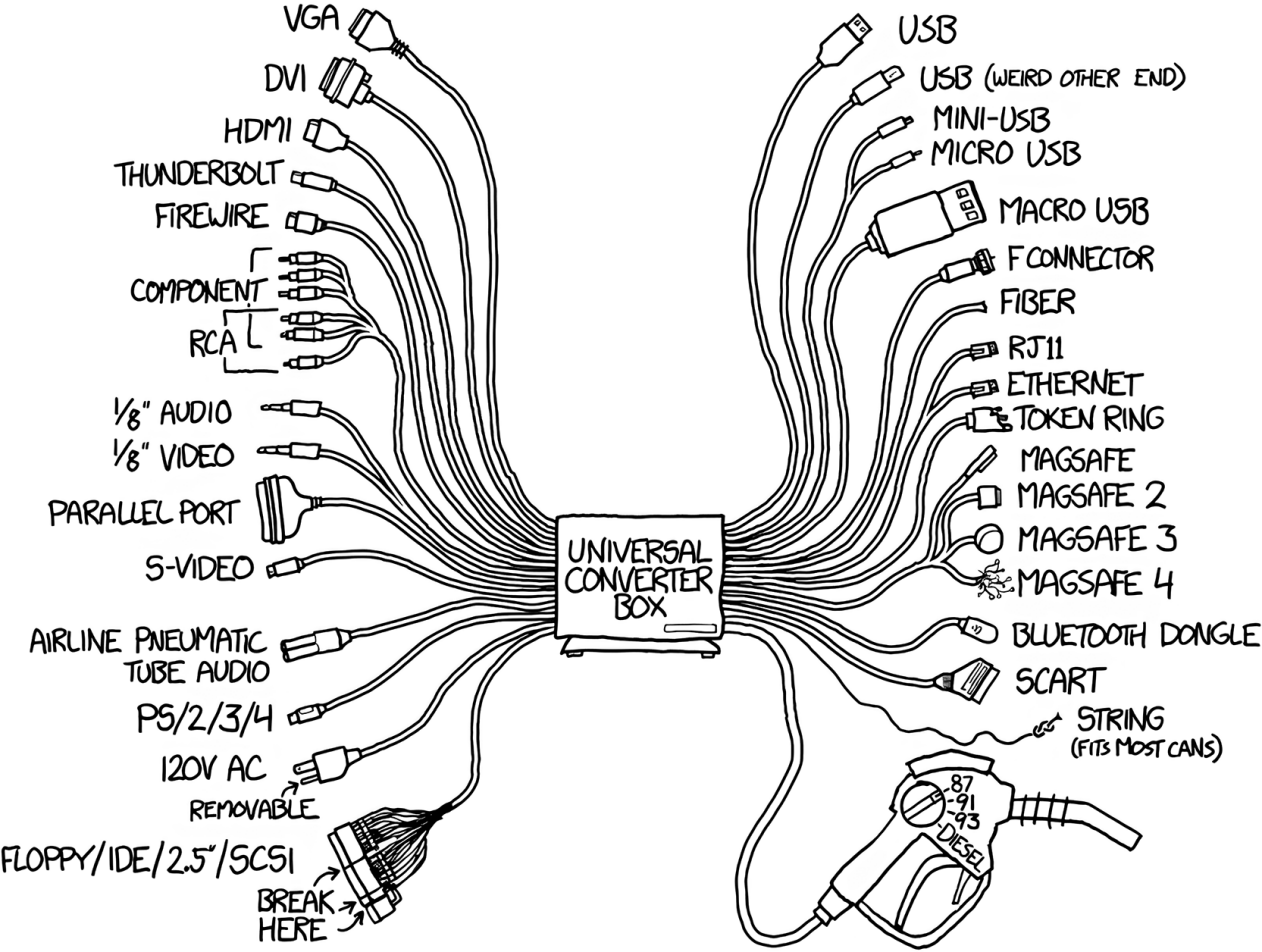
Universal Converter Box by Randall Munroe, illustrating the irony of solving interoperability with yet another standard. © xkcd, licensed under CC BY-NC 2.5. ^22^

The linguistic analysis examined the types of words used to define interoperability and how their usage has evolved. When these definitions are compared to the practical experiences gathered during the int:net project, it becomes evident that many of them tend to emphasize the lower levels of the SGAM model. The analysis revealed that a significant number of definitions focus explicitly or implicitly on the ability of two or more systems to interact. If this interpretation is sufficient, interoperability can be reduced to the technical task of creating adapters between the components. However, this reductionist view does not align with a broader and more intuitive understanding of interoperability. As humorously illustrated by Randall Munroe in one of his comics, such simplistic solutions often fail to address the deeper complexities involved ( Figure 8). ^22^

While this comic is an exaggeration, those who travel or those who forgot their cell phone chargers in the early 2000s will have plenty of real-life experience with adapter wall plugs or dealing with adapters for cell phone chargers before the EU required common chargers through the Radio Equipment Directive. ^15^ When an EU plug does not fit in a wall socket in the UK or North America, there is a lack of interoperability. However, no information was exchanged in this context. Therefore, this example clearly shows that interoperability is not only about the ability to exchange information.

This is a good moment to introduce the related term ‘compatible.’ According to Merriam-Webster, it is defined as: “Designed to work with another device or system without modification”. ^23^ This term is closely related to interoperability by looking at a definition like “Interoperability: Products, systems or organisations are interoperable if they can work together without limitations”. ^24^

At first glance, interoperability and compatibility appear synonymous. However, compatibility is understood more accurately as a dimension of interoperability. This distinction becomes particularly evident in the context of the int:net project, where it was observed that systems intentionally designed to work together exhibit a higher degree of interoperability than those adapted post-hoc for integration.

Designing systems to be compatible, especially when developed by independent organizations, requires the use of standards. However, the mere existence and adoption of standards do not guarantee interoperability. During the early stages of use-case development, it is common to encounter multiple competing standards that emerge simultaneously. For instance, in the domain of remote-controlled components and Internet of Things (IoT) devices, standards such as IEC 61850, OpenADR, and OpenFMB are currently in use. Moreover, in sectors in which components have long operational lifespans, multiple generations of standards often coexist. This is evident in utility operations, where both IEC 60870–5-104 and IEC 61850 are frequently supported for communication between control rooms and substations. In such scenarios, achieving true interoperability requires a gradual convergence among organizations towards a limited set of standards. Without this alignment, the complexity and fragmentation of systems continue to pose significant challenges.

Finally, and perhaps most fundamentally, interoperability is not a quality of a single technology or organization. After all, if two organizations (or technologies) adopt more standards to become more interoperable, but they choose different standards, then those two organizations (or technologies) will still not be interoperable.

Interoperability can only be achieved when adjacent organizations, systems, and technologies adopt the same or compatible standards. This alignment effectively creates a community of interoperable actors and entities that not only adopt similar standards but also develop technologies and methodologies to comply with them. These communities may be highly organized and institutionalized, as seen with the Common Grid Model Exchange Standard (CGMES) ^25^ coordinated through ENTSO-E, or they may emerge more informally, such as through the widespread adoption of specific software tools such as AutoCAD for technical drawings. This introduces a complex dimension to interoperability: it is not solely the result of top-down design but often emerges organically through shared practices and mutual adaptation. As such, interoperability should be understood as an emergent property of a system that arises from the collective behavior and alignment of its participants rather than from centralized planning. Thus, interoperability is an emergent quality of a system. ^14^

Based on the insights gained from the int:net project and the preceding analysis, the following definition of interoperability is proposed:

*“Interoperability refers to the ability of diverse systems, organisations, and individuals to work together seamlessly through shared understanding and mutual compatibility. It is an emergent property that arises from the continuous development and refinement of standards, collaboration among stakeholders, and the dissemination of knowledge across technical, organisational, and societal boundaries.”*

This definition relies on two concepts that deserve highlighting:

• *Compatibility:* Refers to the ability of a device or system to work with another device without requiring modification. This means that technologies are designed to work together, and in the context of organizations, they design/organize themselves to be able to work together.
• *Emergent property:* This is the quality of a complex system that cannot be explained through the qualities of its constituent parts. This is reflected by the observation that one part (technology or organization) cannot be interoperable by itself. Instead, interoperability describes system-wide behaviour.

As interoperability is an emergent property, the qualities like ‘work together seamlessly’ and ‘mutual compatibility’ should be seen as aspirations, or on a spectrum. For instance, if two organizations are attempting to improve their effective collaboration in a value chain through mutually agreed standards and practices, but their collaboration isn’t ‘seamless’ yet, this could still be described as ‘interoperability’.

### 6.2 Implementation recommendations

Achieving interoperability in the energy transition requires a multilayered, iterative, and inclusive approach. As the updated concept extends beyond technical systems to include organizational, societal, and human dimensions, its implementation must reflect this complexity. The following points outline technical, policy, and social guidelines, along with practical strategies, to move beyond static technical compatibility and support the application of this broader definition across the energy ecosystem, drawing on insights from the int:net project:

• **Technical recommendations**

a) *Introduction and usage of Framework Layer:* To implement interoperability as a dynamic and emergent property, existing frameworks must be expanded to address a broader set of interactions. For instance, SGAM has been instrumental in mapping technical interoperability in the energy sector. However, to reflect the redefined concept, SGAM and similar models should be extended or complemented with six-layer accounting for:

i. Social interoperability: Facilitating communication and cooperation between individuals and communities involved in or affected by a specific use case.
ii. Organizational interoperability: Aligning business processes, governance structures, and operational strategies across institutions, policy frameworks, and market dynamics.
This expanded framework can guide stakeholders in assessing gaps, aligning efforts, and prioritizing initiatives that contribute to a cohesive and future-ready energy ecosystem.

b) *Design for adaptability:* Systems must be architected with interoperability-by-design principles, favoring open interfaces, loose coupling, and evolutionary scalability. This allows different components to interoperate without requiring uniformity, and supports the integration of emerging technologies and actors over time.

With adaptability, we are trying to address that, especially in infrastructure with long life cycles, multiple generations of technology operate alongside each other. Standards are often designed in the context of the current state of the art/state of best practice. In the power grid we have seen many examples where new technologies need to interoperate with components from the 70’s or 80’s (before the internet was a thing). And so there becomes a need for inter-generational interoperability. In our experience, the industry has some, but limited experience and know-how for how to design standards to support this. Another factor is that, over such long times, the entire premise on which the infrastructure was designed, has changed. For instance, the switch from centralized generation/decentralized consumption to centralized and decentralized generation/decentralized consumption. The old/analogue power grid supports this to a high degree, but lots of digital components in the grid still assume energy flows from the transmission grid to the distribution grid and not the other way around. To achieve interoperability at the life span of the components of the infrastructure, the standards and technologies need to be designed to adapt to these types of changes.

• **Social recommendations**
  a) *Stakeholder engagement:* A central aspect of implementing holistic interoperability is fostering sustained collaboration across diverse stakeholder groups, including technology providers, utilities, regulators, researchers, and citizens. Co-creation workshops, living laboratories, and participatory design processes can play a vital role in surfacing local knowledge, identifying context-specific challenges, and developing interoperable solutions that are both technically robust and socially acceptable. In this context, the int:net project offers a valuable blueprint for such an engagement, demonstrating how iterative dialogue and joint exploration can reveal limitations in existing interoperability practices and inspire more inclusive approaches.
  b) *Promote interoperability, literacy, and cultural change:* Achieving interoperability requires not only technical alignment but also cultural transformation. This is supported by the inclusion of interoperability concepts in education and training across engineering, social sciences, and governance. This facilitates peer-to-peer knowledge sharing and storytelling across communities to diffuse good practices.
• **Policy recommendations**

a) *Develop flexible standards:* Interoperability must be supported by evolving standards that are not only technically sound but also adaptable to social, organizational, and environmental changes. The effective implementation of this premise requires the following:

i. Ensuring compatibility between international, national, and sectoral standards and avoiding fragmentation that can arise from divergent requirements. Coordination mechanisms such as joint working groups can ensure that priorities are aligned and the implementation is consistent.
ii. Developing modular and flexible standards that accommodate emerging technologies and business models. Standards bodies and regulatory institutions should prioritize stakeholder alignment and the integration of non-technical considerations into standardization processes. This can be operationalized with the deployment of plug-and play components or the definition of interfaces that allow new technologies to integrate seamlessly without requiring re-certification.
iii. Facilitating open access to standards and tools so that actors of all sizes and capacities can participate in energy transition. This involves:

• Ensuring that standards and their toolkits are publicly accessible, removing cost barriers.
• Allowing the sharing of standards related documents
• Allowing technical users to submit issues and contributions without charge

Such measures help democratize access to standardization activities and enable wider participation in the energy transition. However, in practice, achieving open access can be challenging. Many standards provide detailed implementation protocols (e.g., machine-readable files, schemas) that are legally restricted and not freely shareable. These protocols may be licensed under “source-available” agreements that limit modification or redistribution, meaning that even compliant implementations may not be legally treated as fully open source. Addressing these legal and accessibility barriers is essential to ensure that flexible standards can be widely adopted and collaboratively improved.

b) *Capacity building:* A core element of interoperability is shared understanding among the actors. Therefore, implementation must include targeted capacity-building efforts to develop necessary skills, mindsets, and institutional awareness. This includes:

i. Training programs for professionals across disciplines to understand and apply interoperability principles.
ii. Creation of interdisciplinary curricula in higher education that bridges engineering, social sciences, and environmental studies.
iii. Knowledge-sharing platforms that enable real-time exchange of best practices, lessons learned, and emerging insights.

These efforts ensure that interoperability is not just embedded in systems, but in the people and organizations that operate and sustain them.

c) *Monitoring and evaluation:* Given that interoperability is an evolving property, its implementation must be iterative and responsive to feedback. Mechanisms should be put in place to regularly assess interoperability outcomes across technical, social, and organizational dimensions. Identify unintended consequences or gaps. Adapt strategies and standards based on ongoing learning and system development. A flexible monitoring and evaluation framework aligned with the updated definition can help maintain coherence as energy systems evolve and new challenges arise. This monitoring can be conducted by independent third parties or auditors which conduct impartial assessments of technical, social, and organizational interoperability outcomes.
d) *Mandate where appropriate, foster where possible:* The notion of mandating interoperability, or even compliance with specific standards, is a challenging topic. While there are directives that require a certain degree (or scope) of interoperability, such as the Radio Equipment Directive, ^14^ both policymakers and industry are hesitant to comply with specific standards. At best, this was considered heavy-handed. At worst, mandating standards can prevent the industry from addressing new emerging challenges in cases where the solution to those challenges would involve non-compliant technology. However, at this moment, there often seems to be a ‘mandate vacuum’ where no one party or collaborative will take the initiative to decide on a certain standard and push for its adoption, stagnating progress.

A sensible middle might be found in the recognition that interoperability emerges given a new challenge: the industry first needs to try out different solutions, develop an understanding, and make first attempts at solutions. As the industry gains more experience in addressing the challenge, a common understanding emerges, and groups of organizations start to converge towards specific solutions. Only much later, when the same problem has to be solved many times in different contexts, and there is a consensus on best practices and ‘which technology works,’ interoperability emerges. In the first stages of this lifecycle, policymakers should refrain from mandating specific standards and instead focus on supporting innovation, collaboration, and fostering alignment at the industry and project levels to ensure convergence of solutions that have proven successful in specific contexts. It is only during the higher maturity phases of the development that there is broad agreement on how to solve a particular problem, but late adopters of this new technology hold the industry (and society) back, which mandates interoperability or even compliance to a specific standard, might be a valuable addition to the policy landscape.

## 7. Conclusions

This study critically examined the evolving concept of interoperability within the context of energy transition, revealing that these traditional definitions, centered primarily on technical compatibility and information exchange, are no longer sufficient to address the multifaceted challenges of modern energy systems. Drawing on linguistic analysis, historical trends, and practical insights from the int:net project, this study proposes a redefinition of interoperability as a multidimensional and emergent property. This new definition encompasses not only technical systems but also organizational, social, and governance dimensions, reflecting the complex interdependencies that shape energy infrastructures today. The introduction of a sixth “Framework Layer” to the SGAM further underscores the importance of institutional and regulatory considerations in achieving true interoperability.

Future work should focus on operationalizing this definition through the development of assessment methodologies and validation in real-world energy systems. Further research is needed to support the integration of interoperability principles into policy, standardization processes, and interdisciplinary education. These efforts are essential to ensure coherent, adaptive, and inclusive energy system development.

## Declaration of generative AI and AI-assisted technologies in the writing process

The authors used OpenAI during the preparation of this study. (2025). Microsoft Copilot, powered by GPT-4-turbo [a large language model]. https://copilot.microsoft.com, to assist with sentence structure, grammar correction, and stylistic refinement. After using this tool, the authors carefully reviewed and edited all the AI-generated suggestions and took full responsibility for the content and accuracy of the final publication.

## Disclaimer

The views expressed in this article are those of the authors. Publications in Open Research Europe do not imply endorsement by the European Commission.

### Ethics and consent

Ethical approval and consent were not required.

## Data availability

### Underlying data

Zenodo: Definition of Interoperability. https://doi.org/10.5281/zenodo.15724890. ^26^

This project contains the following underlying data:

• **DefinitionsOfInteroperability.xlsx** (Data sheet with the list of different definitions of interoperability from the literature).

Data are available under the terms of the Creative Commons Attribution 4.0 International license (CC-BY 4.0).
