# Supplementary material for: Redefining the concept of interoperability: A multidimensional approach for the energy sector
Source: Open Res Eur. 2026 May 20;5:250. Originally published 2025 Aug 22. [Version 2] doi: 10.12688/openreseurope.20755.2 (PMC12441666; doi:10.12688/openreseurope.20755.2)
Supplement: Peer review history

# Reviewer report: Reviewer response for version 2

**DOI:** 10.21956/openreseurope.25929.r74722

**Published:** 2026-06-08

**Authors:** Eric Lambert

**Affiliations:** 1 EDF – Électricité de France, Paris, France

I approve the paper and thanks to Thomas for having taken my comments.

Is the study design appropriate and does the work have academic merit?

Partly

Is the work clearly and accurately presented and does it cite the current literature?

Yes

If applicable, is the statistical analysis and its interpretation appropriate?

Partly

Are all the source data underlying the results available to ensure full reproducibility?

Partly

Are the conclusions drawn adequately supported by the results?

Partly

Are sufficient details of methods and analysis provided to allow replication by others?

Partly

Reviewer Expertise:

NA

I confirm that I have read this submission and believe that I have an appropriate level of expertise to confirm that it is of an acceptable scientific standard.

# Reviewer report: Reviewer response for version 2

**DOI:** 10.21956/openreseurope.25929.r74724

**Published:** 2026-05-29

**Authors:** Amalia Suryani

**Affiliations:** 1 University of Twente, Enschede, The Netherlands

*Thank you for the opportunity to review the revised version of this manuscript.*

*I have carefully read the revised manuscript and confirm that the authors have appropriately addressed my previous comments and suggestions. The revisions have improved the clarity and overall quality of the work.*

*I have no further comments or concerns. In my opinion, the manuscript is suitable for indexing in its current form.*

Is the study design appropriate and does the work have academic merit?

Yes

Is the work clearly and accurately presented and does it cite the current literature?

Partly

If applicable, is the statistical analysis and its interpretation appropriate?

Partly

Are all the source data underlying the results available to ensure full reproducibility?

Partly

Are the conclusions drawn adequately supported by the results?

Yes

Are sufficient details of methods and analysis provided to allow replication by others?

Partly

Reviewer Expertise:

NA

I confirm that I have read this submission and believe that I have an appropriate level of expertise to confirm that it is of an acceptable scientific standard.

# Reviewer report: Reviewer response for version 2

**DOI:** 10.21956/openreseurope.25929.r74723

**Published:** 2026-05-22

**Authors:** Tharindu Ranathunga

**Affiliations:** 1 Munster Technological University, Cork, Ireland

I don't have any more comments. Thank you for addressing some of my previous suggestions.

Is the study design appropriate and does the work have academic merit?

Partly

Is the work clearly and accurately presented and does it cite the current literature?

Yes

If applicable, is the statistical analysis and its interpretation appropriate?

Partly

Are all the source data underlying the results available to ensure full reproducibility?

Yes

Are the conclusions drawn adequately supported by the results?

Yes

Are sufficient details of methods and analysis provided to allow replication by others?

Partly

Reviewer Expertise:

Data Spaces, Policy Enforcement,  Ontology Design & Data Hormonization

I confirm that I have read this submission and believe that I have an appropriate level of expertise to confirm that it is of an acceptable scientific standard.

# Response

**Published:** 2026-05-22

**Authors:** Thomas STRASSER

**Affiliations:** Center for Energy, Austrian Institute of Technology GmbH, Vienna, Vienna, Austria

Many thanks for your feedback and approval.

# Reviewer report: Reviewer response for version 1

**DOI:** 10.21956/openreseurope.22452.r59045

**Published:** 2025-09-16

**Authors:** Tharindu Ranathunga

**Affiliations:** 1 Munster Technological University, Cork, Ireland

The paper critiques narrow, technical definitions of interoperability and proposes a broader, multidimensional view. Using insights from the Horizon Europe *int:net* project, the authors analyse 40 definitions through lexical and temporal methods and map them onto the SGAM, adding a new “Framework Layer” for governance and institutions. They advance a definition of interoperability as an emergent property shaped by technical, organisational, social, and governance factors, and conclude with technical, social, and policy recommendations to support the energy transition.

 The work is clearly and accurately presented, with arguments that are logically structured and technically precise. It cites relevant and current literature, particularly from the European context, which is appropriate given the project’s scope and funding framework. At the same time, the coverage could be strengthened by pointing to non-European perspectives for context and comparison. For example, references to the *U.S. NIST* Smart Grid Interoperability Framework, *Chinese smart grid standards*, and international *standards from IEC and ISO *would highlight complementarities and reinforce the global relevance of the study.

 The study design is well-suited to a conceptual paper, combining lexical and temporal analysis with SGAM mapping to deliver valuable insights. Its originality lies in the proposed redefinition of interoperability and the addition of an SGAM “Framework Layer,” addressing a timely and underexplored issue with an interdisciplinary lens. However, the dataset is small and Eurocentric, limiting generalizability. In addition, the study would benefit from stronger empirical grounding through case studies, stakeholder interviews, or expert validation.

 The authors provide helpful transparency by outlining how the 40 definitions were gathered and analysed, but some aspects of the methodology could be described in more detail to strengthen reproducibility. For instance, it would be useful to know more about how the definitions were selected, what inclusion or exclusion rules were applied.

 The analysis relies mainly on descriptive methods, such as frequency counts and a scoring system that distinguishes explicit, implicit, and absent references. This is an appropriate approach for an exploratory, conceptually oriented study, and it helps to reveal interesting patterns in the data. The results are presented in a clear and accessible way, with visualisations like word clouds and histograms that make the findings easy to follow. At the same time, the study could be further strengthened by adding a few additional checks. For example, reporting on how scoring consistency was ensured, or incorporating simple statistical tests to support observations about semantic evolution or temporal trends. Even a brief discussion of potential sources of bias or sensitivity in the analysis would make the findings more robust.

 The authors have taken a commendable step by making their core dataset ( *DefinitionsOfInteroperability.xlsx* on Zenodo) openly available, which enhances transparency and allows others to review the underlying material.

 The conclusions are coherent and logically consistent with the results, particularly the finding that interoperability definitions are biased toward technical aspects and underrepresent governance and organisational layers.

 Overall, this paper is timely, well written, and makes a valuable conceptual contribution by broadening the definition of interoperability and extending the SGAM framework to include governance. Its clarity, interdisciplinary scope, and practical orientation are important strengths. To be scientifically sound, however, the article must broaden and update its literature base, expand and diversify its dataset, and provide transparent selection criteria and scoring validation.

Is the study design appropriate and does the work have academic merit?

Partly

Is the work clearly and accurately presented and does it cite the current literature?

Yes

If applicable, is the statistical analysis and its interpretation appropriate?

Partly

Are all the source data underlying the results available to ensure full reproducibility?

Yes

Are the conclusions drawn adequately supported by the results?

Yes

Are sufficient details of methods and analysis provided to allow replication by others?

Partly

Reviewer Expertise:

Data Spaces, Policy Enforcement,  Ontology Design & Data Hormonization

I confirm that I have read this submission and believe that I have an appropriate level of expertise to confirm that it is of an acceptable scientific standard.

# Response

**Published:** 2026-05-08

**Authors:** Thomas STRASSER

**Affiliations:** Center for Energy, Austrian Institute of Technology GmbH, Vienna, Vienna, Austria

We appreciate Reviewer 3's detailed and constructive assessment and address the points raised below. *1. Literature positioning and geographic scope: * While the paper is motivated by insights from the European Horizon Europe int:net project and makes use of SGAM as a European reference framework, the underlying dataset of 40 interoperability definitions is not European centric. As documented in the openly available Excel dataset, the definitions originate from a wide range of domains, sectors, and geographic contexts, including sources from North and South America, international standardisation bodies (IEEE, ISO/IEC, NATO), global industry initiatives, public administrations outside Europe, healthcare, agriculture, defense, and digital economy contexts.

*2. Study design, originality, and empirical grounding:* The study design is deliberately conceptual and exploratory. Its originality lies in the combined use of linguistic analysis, temporal assessment, and SGAM based classification across a cross sector and multinational dataset. While empirical validation through case studies or interviews would provide additional depth, this was beyond the scope of the present contribution and is now more clearly identified as future work. Importantly, the dataset itself already reflects a broad empirical base in terms of sectors and institutional contexts, even if not tied to individual implementation cases.

*3. Methodological transparency and reproducibility:* In response to the reviewer's comments, the manuscript clarifies the definition selection principles, including the decision to rely exclusively on explicit, quoted definitions and to avoid inferred interpretations. The qualitative coding scheme (explicit, implicit, absent references) is now more explicitly motivated, and the complete dataset remains publicly available on Zenodo to support transparency and reproducibility.

*4. Scoring approach, robustness, and bias:* The paper's treatment of the scoring approach remains largely unchanged and continues to reflect its exploratory and descriptive purpose. The scoring system is intentionally used as a heuristic classification tool to compare how different interoperability definitions relate to the SGAM layers, rather than as a statistical or validation instrument. While the revised manuscript includes minor clarifications regarding the purpose of the scoring and the interpretation of Figures 6 and 7, no additional validation measures or statistical consistency checks were introduced. The authors therefore maintain that the method is appropriate for revealing qualitative patterns across definitions, while acknowledging-consistent with the reviewer's observation-that some degree of interpretive subjectivity is inherent to this type of conceptual analysis.

*5. Conclusions and contribution:* The conclusions remain unchanged but are more clearly contextualised. The paper's central contribution is conceptual: demonstrating, across a geographically and sectorally diverse dataset, that prevailing interoperability definitions disproportionately emphasise technical aspects while underrepresenting organisational and governance dimensions. This finding motivates both the proposed redefinition of interoperability as an emergent property and the analytical extension of SGAM with a Framework layer. Overall, the manuscript remains focused on its primary conceptual contribution and has been clarified where necessary to better reflect its scope and methodological intent. Based on a geographically and sectorally diverse dataset of interoperability definitions, the study shows that existing definitions tend to prioritise technical aspects while giving limited attention to organisational and governance-level challenges. These observations motivate the proposed redefinition of interoperability as an emergent property and support the analytical extension of SGAM with a complementary Framework layer, without modifying the original SGAM structure. The paper thus aims to provide a transparent and theoretically grounded basis for further empirical and standardisation-oriented work, rather than a finalized or prescriptive assessment methodology.

# Reviewer report: Reviewer response for version 1

**DOI:** 10.21956/openreseurope.22452.r59044

**Published:** 2025-09-11

**Authors:** Eric Lambert

**Affiliations:** 1 EDF – Électricité de France, Paris, France

Summary of the article : the artcile review the concept and definition of interoperability, and how it evolved. It introduces the SGAM and its 5 interoperability layers, and introduce the need of a sixth layer (Framework). 

**Main remark:** The article cites the **GWAC** layers (reference 7 is not accurate ...) but could precise that SGAM was a simplification of GWAC. It miss some external references from IEC describing SGAM (IEC 63200), freely available (on IEC SyC Smart Energy web page IEC SRD 63200 - SGAM basics - SyC Smart Energy), This webpage shows clearly the mapping between GWAC and SGAM...

 The Framework layer is introduced but a clear example should be proposed to assess its real need. SGAM is known but not use as it should be... Is the 6th layer will help to use SGAM more efficiently? **Do the authors want to update IEC 63200 with this new layer?**

 Remarks on the article

 1. these 2 sentences are quite the same: The introduction of smart grids in the late 1990s further highlighted the importance of standardized protocols for integrating information and communication technologies with traditional energy systems ^2^. The late 1990s saw the emergence of the smart grid concept, underscoring the importance of standardized protocols for integrating information and communication technologies with traditional energy systems ^3^

^2. reference 7 is not reachable^

^3. §3.1 in standardisation (like at IEC), terms are defined with an explicit definition. For instance, the article coud refer to Electropedia, which propose definitions of interoperability :^

^4. §3.2 : this § could also leverage and refer to IEC report on semantic interoperability: :^ New IEC Report on semantic interoperability

Semantic interoperability: challenges in the digital transformation age | IEC

 5. §4.3 In the smart grid domain as TC57 is developping 2 Core standards series for information exchange/interoperability (CIM and 61850), it could also refer to the **name** of IEC TC 57 technical committee, which has not changed over 30 years... : **Power systems management and associated information exchange**. This has not changed and probably  not be changed yet, as it is still relevant...

 6.  §4.4 : Electropedia which is a free IEC standard, has also its definitions. It does not prevent standard document to precise their "interopérability" concept, by using several words "semantic interoperability", "interoperability profile" : see definition at https://sepapower.org/knowledge/interoperability-profiles-a-better-way-to-buy-grid-technology/

 We could also argue that for some complex area,  a simple definition is not sufficient. For instance see this reference:  https://www.pacw.org/interoperability 

 And again, associated terms can be more meaning full : "61850 interoperability tests" which leads to https://www.pacw.org/iec-61850-interoperability-tests among others.

 7. I'm a bit puzzled with this 6th Layer as the SGAM figure 4  in IEC 62913-1 is refering to  Business Objectives (political, Regulatory, Framework)...

 See : IEC SRD 63200 - SGAM basics - SyC Smart Energy which should be mentioned also in §5

 The SGAM layers are based on  GWAC layers and the Business layer includes Polotical/regulatoryu aspects...  

 8. In the usage of SGAM, by IEC 62913-1, (see figure 5) Business Roles (extracted for instance from HEMRM Harmonized Electricity Market Role Model) in Europe, are at the BUsiness Level layer, as technical actors (like a SCADA, a gateway, are at the component layer, and are associated to systems/functions defined at the Function Layer. As a consequence I'm not sure that "However, the simultaneous consideration of both technical and organizational actors, as exemplified by the SGAM component layer, is not always observed." is a right assumption as organizational actors are at the business layer level.

 9. Spanish definition is below figure 7, prefix the definition "Spanish definition of interoperability".

 10. The SGAM usage is directly linked to Use case definitions (either business use cases, and system use cases, as defined in IEC 62913-1). it then helps to fulfill the use case template with captured requirements and references to regulatory obligations/ Business constraints / ...

 11. You could refer to  Interoperable Europe act

Interoperable Europe Act | Interoperable Europe Portal and this reference : Interoperability Architecture Solutions | Interoperable Europe Portal which refer to Structural Interop., Behavioural Interop, Governance interop...

 12. 6.2 : the recommendation is in fact introducing  some of the GWAC layers... The main idea of SGAM was to simplify the GWAC... 

 13. Conclusion : the main recommendation is as written to " **operationalizing interoperability**". It may be recommended to set up regular interoperability tests, and to have clear certification schemes.

 The ENTSO-E CGMES is a very good example (keeping in mind that moving a CGMES version to another is not an easy task...utiliies and vendors need to be convinced...). Another "success" is  the 61850 regular IOP tests organized annually. The problem is that interoperabiloty platforms should be up and running 24/24 7/7 in order to  conduct tests on a regular basis...

**Do the authors want to update IEC 63200 with this new layer? is it a top priority in the context of Interoperability?**

Is the study design appropriate and does the work have academic merit?

Partly

Is the work clearly and accurately presented and does it cite the current literature?

Yes

If applicable, is the statistical analysis and its interpretation appropriate?

Partly

Are all the source data underlying the results available to ensure full reproducibility?

Partly

Are the conclusions drawn adequately supported by the results?

Partly

Are sufficient details of methods and analysis provided to allow replication by others?

Partly

Reviewer Expertise:

Smart Grid standardisation.

I confirm that I have read this submission and believe that I have an appropriate level of expertise to confirm that it is of an acceptable scientific standard, however I have significant reservations, as outlined above.

# Response

**Published:** 2026-05-08

**Authors:** Thomas STRASSER

**Affiliations:** Center for Energy, Austrian Institute of Technology GmbH, Vienna, Vienna, Austria

We appreciate Reviewer 2's detailed and constructive assessment and address the points raised below.

*ad Main Remark:*

 We acknowledge the relationship between GWAC and SGAM and clarify this more explicitly in the revised manuscript. As reflected in the updated text, SGAM is treated as the established baseline for analysing interoperability challenges, and the paper systematically classifies existing interoperability definitions against the SGAM layers, including the extended sixth "Framework" layer. In line with our response to Reviewer 1, the paper does not modify or reinterpret the original SGAM layers; rather, it introduces the Framework layer as an analytical extension to capture governance-level aspects that consistently operate above individual business cases. No further changes to the core SGAM concept were made beyond this clarification. Furthermore, the Framework layer is introduced to explicitly capture institutional and stakeholder frameworks, such as regulation, standardisation, and funding, that influence interoperability across domains but are only implicitly addressed in current SGAM applications. This supports a more effective use of SGAM in cross-sector and system-of-systems contexts without modifying the original five layers. The term Framework is deliberately chosen over Governance to reflect the coexistence of multiple regulatory and institutional frameworks shaping governance relationships. The authors do not propose an update to IEC 63200; the layer is presented solely as a complementary analytical extension, consistent with the int:net D4.3 and D4.4 deliverables (see https://intnet.eu/resources).

*ad Point 1:*

 The two sentences indeed conveyed the same content. In the revised manuscript, we have removed the redundancy by merging them into a single sentence, thereby improving clarity and avoiding repetition while preserving the intended historical context and citation support.

*ad Point 2:*

 The original document is not available anymore. We have replaced reference 7 with the related standard document ISO/IEC TR 15067-3-2:2016: Information technology - Home electronic system application model - Part 3-2: GridWise - Interoperability context-setting framework.

*ad Point 3 & 4:*

 Section 3 mainly addresses language, meaning, and transformation in general over time, which also reflects the term interoperability. Especially, Section 3.1 addresses the triangle of meaning and Section 3.2. semantic changes over time. Standards and their definitions are not directly part of the discussion in this section. Anyhow, ISO/IEC 2382 Information Technology Vocabulary is already included in our survey and the linked dataset. We have therefore addressed this point already.

*ad Point 5 & 6:*

 It is not clear how exactly this remark is intended (seemingly relevant to Section 4.3, nevertheless, not clear). The remark could be fitting for Section 4.2, where some definitions are presented, but the name of a TC is not the same as a definition. While the collection of definitions (unfortunately) doesn't include one from TC57, it does include one from the IEC. All in all, the remark has led to the addition of a line of text at the end of Section 4.4.

*ad Point 7 & 8:*

 Among other reasons, we saw that the 5th layer commonly fell short of systematically incorporating relevant societal considerations, such as institutional arrangements and coordination-relevant requirements, thus failing to consider some stakeholders' perspectives, particularly relevant for cross-sector integration, and hence to provide sufficient context for a specific innovation.

*ad Point 9:*

 The paper has been updated accordingly.

*ad Point 10:*

 The authors agree that SGAM is closely linked to use-case development as defined in IEC 62913-1 and that it effectively supports the structured capture of requirements, business constraints, and regulatory obligations within use-case templates. This paper does not aim to replace or revise this established use-case-driven application of SGAM. Instead, it builds on it by using SGAM as an analytical lens to assess how interoperability definitions address challenges across layers. The proposed Framework layer is intended to complement this usage by making higher-level, cross-use-case conditions-such as regulatory alignment, institutional coordination, and framework-setting constraints - explicit, thereby enriching the context in which business and system use cases are specified rather than altering the existing SGAM-use-case relationship.

*ad Point 11:*

 That is an interesting suggestion, and we did look at the Act. However, to our surprise and disappointment, it does not contain an explicit definition of interoperability; only the term 'cross-border interoperability' is defined. We decided to only take direct quotes for the definition, and the emphasis here is on the cross-border aspect, so we felt it was not a good candidate for our analysis.

*ad Point 12:*

 Absolutely correct, the introduction of this layer is not original work from this paper; we are just referring to one of the other outcomes of the project. Having said that, the reason the framework layer was introduced is closely related to the idea that an organization has a degree of agency, and interoperability is about multiple such agents coming together, so there is an aspect in which any 1 organization has insufficient agency/mandate to affect a successful, interoperable outcome. So, the project members felt it appropriate to represent that as a separate layer.

*ad Point 13:*

 Those are good suggestions of successful examples. Standardization and testing are tried and true methods for achieving interoperability, but those are beyond the scope of this paper. If you are interested, the int:net project has published several documents that go very deep into these subjects (see https://intnet.eu/resources/), including: (i) standardization matrix, (ii) interoperability maturity models and assessments, and (iii) several papers on testing, including harmonization of procedures. *Final remarks on IEC 63200:* The authors do not propose an immediate update to IEC 63200, nor do they present this as a top priority. Rather, this work is intended to open the topic for discussion, informed by the systems-of-systems challenges observed in the int:net project. The proposed Framework layer is presented as a complementary analytical concept that may help address governance- and coordination-related interoperability issues that increasingly arise in complex, cross-domain energy systems. Whether and how such an extension should be reflected in future standardisation activities, such as IEC 63200, is deliberately left as an open question for further debate and community consideration.

# Reviewer report: Reviewer response for version 1

**DOI:** 10.21956/openreseurope.22452.r59041

**Published:** 2025-09-08

**Authors:** Amalia Suryani

**Affiliations:** 1 University of Twente, Enschede, The Netherlands

Thank you for inviting me to review this insightful paper. This study makes a valuable contribution to the field of energy interoperability, particularly by offering a critical examination of how interoperability is defined. I appreciate the authors' efforts to deep dive into the linguistic dimension of the concept, which strengthens the theoretical foundations of interoperability. The article presents a refreshing perspective that goes beyond the technical view of interoperability. 

 I have several questions and comments outlined below:

1. The process of selecting the 40 definitions is not sufficiently explained in the Method section. How was the scope determined? From the available data, the health sector was not represented, whereas the agriculture sector is included.
2. During data cleaning, the authors note that "words that have very similar meaning" were combined. Since similarity is subjective, it would be useful to provide an annex or supplementary material listing these words.
3. Given that the paper considers the use of interoperability across different sectors, it would be valuable to compare how frequently the term is used in different domains. Is the energy sector the dominant one, or perhaps health?
4. Have the authors considered calling the "Framework" layer as "Governance" layer? If so, what motivated the choice of "framework"? "Governance" gives a more straightforward meaning.
5. Did the authors intend to modify the current elements of SGAM layers rather than only add a new one? For instance, the original "Business" layer already includes political and regulatory frameworks. If revisions to SGAM beyond adding a new layer are proposed, this should be stated explicitly.
6. Figures 6 and 7 are unclear. What is the purpose of assigning these scores, and how were they obtained? A methodological description should be added.
7. Please clarify, is it a typo in Section 6.1: Should "Internet project" be corrected
8. The narrative in the second paragraph in Section 6.1. seems to describe compatibility rather than interoperability. The suggested reference illustrates the difference between the two.
9. If interoperability implies working together seamlessly without modification, does this require all energy systems to have a "plug-and-play" feature? Or is some degree of modification acceptable?
10. The technical recommendations would benefit from concrete steps. For example, should "plug-and-play" be the ultimate goal of interoperability? What specific technical objectives should be prioritised? What does "adaptability" entail in practice?
11. Similarly, the policy recommendations could be made more concrete, particularly regarding "develop flexible standards." What does "facilitating open access to standards" mean in practice, given that standard development typically involves multiple stakeholders (often businesses) and is a lengthy process?
12. Who should be responsible for carrying out monitoring and evaluation activities?

Reflecting on my own work on interoperability, I suggest the authors consider citing the following three publications, which could provide additional context.

- A book chapter discussing SGAM, which may be particularly relevant for Section 5 and the proposal to add a new layer.
- A systematic literature review focusing on microgrid interoperability that critiques the limitations of ICT-focused definitions. This source clarifies the distinction between interoperability and compatibility.
- An empirical paper showing how different stakeholder interpretations of interoperability lead to different priorities. These findings could enrich Sections 2 and 6, especially regarding the impact of ambiguous terms, discussion on standards, and recommendations.

I look forward to the authors' responses and to seeing this work indexed after revision.

Is the study design appropriate and does the work have academic merit?

Yes

Is the work clearly and accurately presented and does it cite the current literature?

Partly

If applicable, is the statistical analysis and its interpretation appropriate?

Partly

Are all the source data underlying the results available to ensure full reproducibility?

Partly

Are the conclusions drawn adequately supported by the results?

Yes

Are sufficient details of methods and analysis provided to allow replication by others?

Partly

Reviewer Expertise:

Energy access, interoperability, microgrid, renewable energy, co-creation, socio-technical systems, stakeholder theory, technology management

I confirm that I have read this submission and believe that I have an appropriate level of expertise to confirm that it is of an acceptable scientific standard, however I have significant reservations, as outlined above.

## References

[1] : The Use Case and Smart Grid Architecture Model Approach. 10.1007/978-3-319-49229-2 10.1007/978-3-319-49229-2

[2] : Interoperability in Microgrids to Improve Energy Access: A Systematic Review. *IEEE Access* .2024;12: 10.1109/ACCESS.2024.3396275 64267-64284 10.1109/ACCESS.2024.3396275

[3] : Stakeholder Perspectives on Microgrid Interoperability in Energy Access. *IEEE Access* .2025;13: 10.1109/ACCESS.2025.3587347 119362-119379 10.1109/ACCESS.2025.3587347

# Response

**Published:** 2026-05-08

**Authors:** Thomas STRASSER

**Affiliations:** Center for Energy, Austrian Institute of Technology GmbH, Vienna, Vienna, Austria

We appreciate Reviewer 1's detailed and constructive assessment and address the points raised below.

*ad Point 1: * For the collection method, the authors searched as many definitions of interoperability as they could find in public documents. We focused on collecting definitions from multiple languages that the authors speak well to see if there were any cultural influences. This method certainly has a degree of randomness to it. One aspect that made it harder was that many sources we studied do not actually define the term. A great example is the Interoperable Europe Act, which includes a section on definitions, but the term 'interoperability' itself is not defined, only 'cross-border interoperability'. This was a recurring theme. We decided not to infer any definition from any of the texts but only take direct quotes. We do have, in fact, 1 healthcare-based definition on interoperability, in row 32 of the Excel.

*ad Point 2:* The combined words have been added to the Excel attachment containing the definitions on which the analysis is based.

*ad Point 3:* This would indeed be interesting, but it would require a (statistically representative) corpus of all the documents from these sectors, and that was simply beyond the scope of this paper.

*ad Point 4:* The authors do not intend to modify or redefine the existing SGAM layers. While we fully acknowledge that the original Business layer already encompasses political, regulatory, and market-related aspects, the contribution of this paper, consistent with the int:net D4.3 and D4.4 deliverables (see https://intnet.eu/resources), is the explicit addition of a sixth "Framework" layer without altering the scope of the original five layers. The rationale is that the Business layer mainly addresses business objectives and processes tied to specific use cases, whereas governance-level aspects such as inter-institutional coordination, cross-sector framework setting, alignment of regulation, standardisation, funding, and validation consistently operate above individual business cases. The proposed Framework layer, therefore, complements SGAM by making these institutional and stakeholder interactions explicit and analysable, particularly for system-of-systems and cross-domain interoperability challenges, rather than revising or replacing any existing SGAM layer.

*ad Point 5:* The authors do not intend to modify or redefine the existing SGAM layers. While we fully acknowledge that the original Business layer already encompasses political, regulatory, and market-related aspects, the contribution of this paper, consistent with the int:net D4.3 and D4.4 deliverables, is the explicit addition of a sixth "Framework" layer without altering the scope of the original five layers. The rationale is that the Business layer mainly addresses business objectives and processes tied to specific use cases, whereas governance-level aspects such as inter-institutional coordination, cross-sector framework setting, alignment of regulation, standardisation, funding, and validation consistently operate above individual business cases. The proposed Framework layer, therefore, complements SGAM by making these institutional and stakeholder interactions explicit and analysable, particularly for system-of-systems and cross-domain interoperability challenges, rather than revising or replacing any existing SGAM layer.

*ad Point 6:* The underlying idea of the analysis was to examine the extent to which the collected definitions address the various interoperability challenges. The SGAM framework serves as a solid foundation for systematically identifying these challenges. Furthermore, we consider the inclusion of the sixth SGAM layer development essential for capturing additional aspects of interoperability (see related questions). The methodology for deriving the points was refined, and the purpose was added to the text to enhance clarity.

*ad Point 7:* Yes, this is a typo. The correct term is "int:net project".

*ad Point 8:* The authors agree with this observation and have revised Section 6.1 to more clearly distinguish compatibility from interoperability. The revised text now explicitly frames compatibility as a necessary but insufficient condition for interoperability and clarifies that describing systems that work together "without modification" corresponds to compatibility, whereas interoperability is understood as a broader, emergent system property that also depends on organizational alignment, shared standards, governance arrangements, and ongoing coordination. This clarification aligns the narrative with the conceptual distinction discussed in the literature and ensures that the section no longer conflates the two terms.

*ad Point 9:* As interoperability is also an emergent property, these qualities should indeed be considered as on a spectrum. We have updated the paper to clarify this point.

*ad Point 10:* At this level of abstraction, it is impossible to make meaningful statements about concrete steps that apply everywhere. The nature of standardization within different contexts is just too diverse. Plug and play is certainly a good goal to aim for, but in the complexity (in the complex adaptive sense), priorities may change over time, and plug-and-play may prove to be too high a goal to add value. In that sense, interoperability is just another means to an end. The term adaptability deserves more explanation, and this will be added to the paper.

*ad Point 11:* We appreciate this comment. The recommendations remain intentionally broad at this stage, as their specificity depends on how the newly redefined concept of interoperability is implemented in practice. Nevertheless, we have clarified the concepts highlighted and updated the paper accordingly. Regarding the policy recommendation to "develop flexible standards," we now elaborate on what "facilitating open access to standards" entails in practice. Namely, promoting transparency, inclusiveness, and accessibility in the standards development process, encouraging participation from a wider range of stakeholders (including non-commercial actors), and ensuring that resulting standards are publicly available without restrictive licensing barriers.

*ad Point 12:* This monitoring can be conducted by independent third parties or auditors who conduct impartial assessments of technical, social, and organizational interoperability outcomes. *Final remarks on literature suggestions:* The reference by Gottschalk et al. has been included in the revised manuscript, as it aligns well with the paper's scope and strengthens the discussion of SGAM and its use-case-driven application in Section 5. The two papers by Suryani et al. (2024, 2025) were not included, as their focus is on microgrids and energy access, whereas this paper deliberately addresses interoperability from a broader, system-of-systems perspective across the energy sector.
